# Brightfield multiplex immunohistochemistry with multispectral imaging

**DOI:** 10.1038/s41374-020-0429-0

**Published:** 2020-04-27

**Authors:** Larry E. Morrison, Mark R. Lefever, Lauren J. Behman, Torsten Leibold, Esteban A. Roberts, Uwe B. Horchner, Daniel R. Bauer

**Affiliations:** 1 Roche Tissue Diagnostics (Ventana Medical Systems, Inc.), 1910 E Innovation Park Drive, Tucson, AZ 85755 USA; 2 Roche Tissue Diagnostics (Ventana Medical Systems, Inc.), 2841 Scott Blvd, Santa Clara, CA 95050 USA

**Keywords:** Preclinical research, Imaging the immune system, Cancer imaging

## Abstract

Brightfield microscopy is the preferred method of pathologists for diagnosing solid tumors, utilizing common staining techniques such as hematoxylin and eosin staining and immunohistochemistry (IHC). However, as our understanding of the complex tumor microenvironment grows, there is increasing demand for multiplexed biomarker detection. Currently, multiplexed IHC assays are almost exclusively based on immunofluorescence because brightfield techniques are limited by the broad spectral absorption of chromogens and a reliance on conventional 3-channel color cameras. In this work, we overcome these limitations by combining new chromogens possessing narrow absorbance bands with matched illumination channels and monochrome imaging. Multiplex IHC was performed using four or five covalently deposited chromogens and hematoxylin nuclear stain to preserve morphological context and detail. Brightfield illumination was provided with a tungsten lamp/filter wheel combination or filtered light emitting diodes to provide up to 12 illumination wavelengths. In addition, an automated rapid imaging system was developed, using a synchronized 12-LED illuminator, that could capture images at all wavelengths in under 1 s. In one example, a four-biomarker multiplex assay was designed and used to distinguish regions of adenocarcinoma and squamous cell carcinoma in non-small cell lung cancer. The technology was also validated with a five-biomarker assay in prostate cancer. Spectrally unmixed images of each biomarker demonstrated concordant expression patterns with DAB single stain on serial sections, indicating faithful identification of each biomarker. In each assay, all chromogens were well resolved by spectral unmixing to remove spectral crosstalk. While further characterization and refinement of the assay, and improvements in automation and user interface are necessary for pathologist acceptance, this approach to multiplex IHC and multispectral imaging has the potential to accelerate adoption of multiplexing by combining the medical value of high-order multiplexing with the speed, pathologist familiarity, and broadly established clinical utility of brightfield microscopy.

## Abstract (web summary)

Brightfield multiplex immunohistochemistry (IHC) is improved by replacing broadly absorbing chromogens with narrowband covalently deposited chromogens, and sequentially illuminating with light channels matched to chromogen absorbance bands, synchronized with monochromatic image acquisition. Light emitting diodes provide a path to rapid multispectral imaging. Spectral unmixing provided accurate representations of biomarkers that faithfully reproduced 3,3′-diaminobenzidine IHC.

## Introduction

Brightfield microscopy is the current clinical standard for cancer diagnostics and has long been the choice of pathologists for interpreting suspect tissues, with hematoxylin and eosin (H&E) stain and immunohistochemistry (IHC) being mainstays for clinical diagnostics in solid tumors and cytology specimens [1, 2]. This preference for brightfield microscopy is partly historical since stains such as H&E have been in medical use for well over a century [1]. Many different stains with different tissue specificities have been developed and pathologist training has concentrated on interpreting these brightfield stains and their highly nuanced staining patterns that reflect minute alterations in cellular and tissue morphology associated with disease [3]. Targeted chromogenic staining of specific tissue components on a molecular level via IHC has greatly augmented H&E leading to much improved diagnosis, prognosis, and prediction of therapeutic response [2]. In addition to the volume of pathologic knowledge associated with brightfield techniques, the higher light levels in brightfield microscopy lead to lower equipment costs, less controlled settings, and shorter image acquisition times relative to immunofluorescence (IF) microscopy.

Nearly all clinical IHC is currently performed to detect a single biomarker per slide-mounted specimen. Detecting more than one biomarker per specimen slide, termed multiplexing, has important advantages, including conserving precious specimen. Multiplexing also permits direct observation of different cell populations within a specimen, termed digital phenotyping, which enables mining of the cellular spatial context within the tumor microenvironment for correlation with patient outcome. Specific cell populations are identified by cell type-specific biomarkers or by the combination of expressed biomarkers and the cellular compartments in which they are expressed. Identification of cell location and cell-to-cell and cell-to-tumor boundary distances have been of particular importance in understanding the function of immune cells in controlling tumor growth and the effectiveness of drugs targeting the interaction between immune and tumor cells [4].

Despite clinical IHC being predominantly brightfield-based, multiplexing is typically performed using IF [5, 6] since multiplexing in brightfield has been problematic. These problems are two-fold. First, relative to the many fluorophores available for IF, there are relatively few chromogens and their absorbance spectra are typically broad compared with fluorophore absorbance and emission spectra. Common chromogens have absorbance peaks with full width at half maxima (FWHM) of 200 nm or more (e.g., 3,3’-diaminobenzidine and Fast Red) compared with fluorophores with FWHM typically between 30 and 60 nm in solution, with some broadening when deposited [7]. This means that two or three chromogens in combination may fill the entire visible spectrum, with considerable spectral overlap between chromogens.

The second problem facing brightfield microscopy is that commercial color cameras only detect three broadly defined colors (red, green, and blue) which are fixed spectrally to reproduce human vision. This limits the number of dyes that can be completely resolved, including potentially overlapped dyes due to biomarker coexpression, to three, and requires that these three dyes have spectral characteristics compatible with the three-color channels in an RGB camera. Fluorescence imaging is performed typically with a monochrome camera combined with multiple filter sets that match the absorbance and emission characteristics of each fluorophore. This allows imaging and resolution of as many fluorophores as spectral separation permits with the disadvantages that time is required to change between filter sets and separately collect and process the different filtered images.

**Fig. 1:**
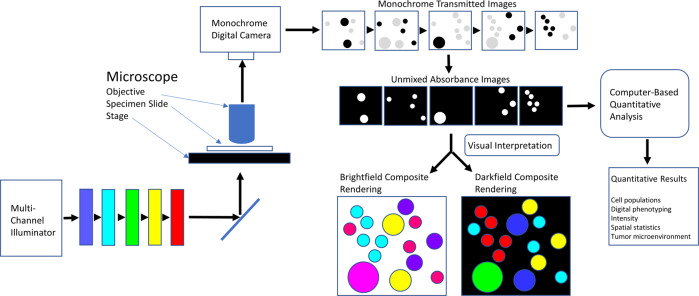
Schematic of multispectral brightfield imaging system. Specimen stained by multiplex IHC is illuminated squentially with multiple light channels matched to the absorbance bands of the chromogens and synchronized with imaging of the transmitted light on the monochrome camera. Images are unmixed and used for quantitative analysis or contruction of composite images for visual interpretation.

Given the preference of brightfield IHC among pathologists, we have addressed the deficiencies of brightfield IHC by developing a system with the speed and user familiarity of brightfield microscopy with the higher-order multiplexing capacity of IF. To address the first problem, we employ covalently deposited chromogens (CDCs) that rely on enzymatic activation of dyes conjugated with tyramide [7] and quinone methide precursors [8] to produce stains covalently bound to cellular and tissue components surrounding the sites of targeted proteins. CDCs have the significant advantage of rapid and facile development of new chromogens with desired spectral characteristics, thereby permitting development of chromogen stains with narrow and well-separated absorbance bands, similar to fluorophores [7]. To address the second problem, we employ a monochrome camera and sequential illumination with narrow bands of illumination matched to the absorbance bands of the discrete chromogens. A schematic of this approach is shown in Fig. 1. Illumination is provided by a filter wheel fitted with narrow bandpass filters in combination with a continuous light source, or by a collection of light emitting diodes (LEDs) paired with narrow bandpass filters, for further spectral definition. Coordinated rapid LED pulsing and image acquisition provides the basis for high-speed multiplex imaging. While this approach requires optical filtering, like fluorescence, or LEDs, the equipment is simplified relative to fluorescence by not requiring epi-fluorescence optics, dichroic mirrors, and emission filters. Brightfield measurements are considerably faster than fluorescence since fluorescence light intensities are much weaker than transmitted light. Still, pathologists will need to accept the additional hardware, electronics, and software needed to achieve the higher levels of multiplexing in brightfield. With this approach, clinically relevant four- and five-chromogen IHC plus hematoxylin counterstaining is demonstrated on non-small cell lung cancer (NSCLC) and prostate tumor specimens.

## Materials and methods

### Specimens

Formalin-fixed paraffin-embedded (FFPE) slide-mounted sections from prostate tumor of anonymized patients were obtained from the Roche Tissue Diagnostics biobank. An anonymous FFPE slide-mounted specimen with adenosquamous carcinoma of the lung was obtained from the archive of Thomas Jefferson University Hospital, Department of Pathology Anatomy and Cell Biology, after obtaining the appropriate IRB approval [9].

### Primary antibodies and detection reagents

**Fig. 2:**
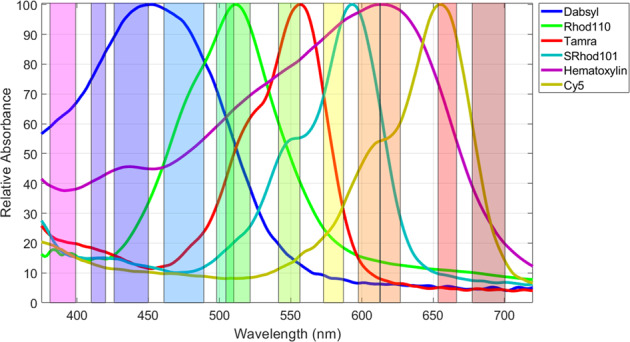
Spectral profile of chromogenic library and 12 channel light engine. Relative absorbance spectra of covalently deposited chromogens and hematoxylin with overlays of the 12 filtered LED illumination channels. Each light channel is represented as a rectangular region with width equal to the light channel’s FWHM.

Primary antibodies, detection reagents, and IHC instruments were obtained from Ventana Medical Systems, Inc. (Tucson, AZ). Primary antibodies for differentiating adenocarcinoma (Ad Ca) and squamous cell carcinoma (SCC) in NSCLC specimens included anti-p40 (BC28, VMSI Cat# 790–4950), anti-cytokeratins 5 & 6 (CK5/6) (D5/16B4, VMSI Cat# 790–4554), anti-Thyroid Transcription Factor-1 (TTF-1) (SP141, VMSI Cat# 790–4756) and anti-Napsin A (MRQ-60, VMSI Cat# 760–4867). Primary antibodies for prostate targets included anti-alpha-methylacyl-CoA racemase (P504S) (SP116, VMSI Cat# 790–6011), anti-CD8 (SP57, VMSI Cat# 790–4460), anti-erythroblast-transformation-specific-related gene (ERG) (EPR3864, VMSI Cat# 790–4576), anti-basal cell (cocktail of antibodies to p63 and keratin; 34ßE12 + p63, VMSI Cat# 790–4536), and anti-Ki-67 (30–9, VMSI Cat# 790–4286). Chromogenic reagents included 4-(4-dimethylaminophenylazo)benzensulfonyl- (dabsyl), carboxytetramethylrhodamine- (TAMRA), and Cy5-based CDCs, available commercially in the DISCOVERY RUO Yellow kit (Cat. No. 760–239), DISCOVERY RUO Purple kit (Cat. No. 760–229), and DISCOVERY RUO Teal kit (Cat # 760–247), respectively. These kits also include the requisite enzyme-antibody conjugates. Methods for synthesizing the rhodamine 110 (Rhod110) and sulforhodamine 101 (SRhod101) tyramide CDCs were described previously [7]. The peroxidase-antibody conjugates used with these CDCs were OmniMap anti-Ms HRP (RUO), DISCOVERY (VMSI Cat# 760–4310), OmniMap anti-Rb HRP (RUO), DISCOVERY (VMSI Cat# 760–4311), UltraMap anti-Ms Alk Phos, DISCOVERY (VMSI Cat# 760–4312), and UltraMap anti-Rb Alk Phos, DISCOVERY (VMSI Cat# 760–4314). Spectra of CDCs deposited individually on tonsil FFPE specimens and targeting Ki-67 are plotted in Fig. 2, and were recorded as previously described [7].

### Immunohistochemistry

Fully automated multiplexed detection was performed on a DISCOVERY Ultra system using the above primary antibodies and detection reagents. Single marker DAB staining was accomplished using a Benchmark XT or Benchmark Ultra system and the ultraView Universal DAB Detection Kit (Cat. No. 760–500), according to the manufacturer’s recommendations. The DISCOVERY Universal Procedure was used to create a protocol for the 4- and 5-multiplex IHC plus hematoxyin. In general, multiplex IHC was performed at 37 °C, unless otherwise noted, as follows (with exceptions specified in commercial detection kits). A slide-mounted paraffin section was deparaffinized by warming the slide to 70 °C for 3 cycles each 8 min long. Antigen retrieval was performed by applying Cell Conditioning 1 (VMSI Cat# 950–124) and warming the slide to 94 °C for 64 min. Staining of each biomarker was performed in sequential steps that included incubation with primary antibody targeting that biomarker, washing in reaction buffer (10x concentrate, VMSI Cat#950–300) to remove unbound antibody, incubation with antispecies antibody targeting the primary antibody (either antimouse or antirabbit) conjugated to either peroxidase or alkaline phosphatase, depending on whether the chromogen is a tyramide or quinone methide derivative, respectively, washing with reaction buffer, incubation with tyramide or quinone methide precursor chromogenic reagent, and washing with reaction buffer. Before the application of the next biomarker in sequence, the slide was incubated with Cell Conditioning 2 (VMSI Cat# 950–123) at 100 °C for 8 min., followed by washing in reaction buffer. Slides were then counterstained with diluted Hematoxylin II (VMSI Cat# 790–2208) and Bluing (VMSI Cat# 760–2037) for 4 min and washed with reaction buffer. Slides were then manually dehydrated through an ethanol series (2 × 80% ethanol, 1 min each, 2 × 90% ethanol, 1 min each, 3 × 100% ethanol, 1 min each, 3× xylene, 1 min each), at ambient temperature, and mounted in Cytoseal xyl (ThermoFisher Scientific, Waltham, MA).

### Microscopes and imaging systems

**Fig. 3:**
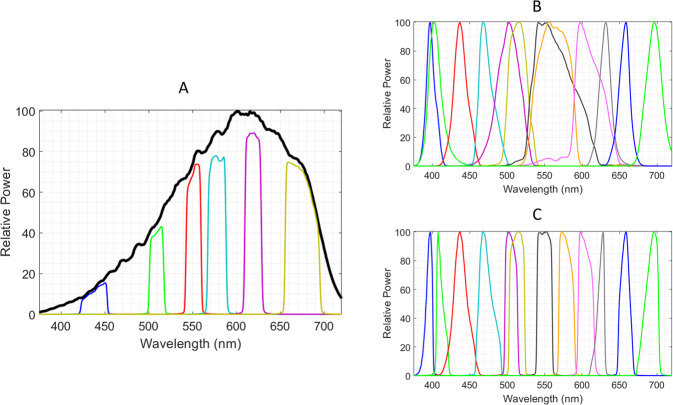
Relative power spectra of illumination channels. **a** Illumination spectra of 100 W tungsten microscope lamp (solid black line) in addition to individual light channels produced after bandpass filtering. **b** Illumination spectra of 12-LED illuminator without filtering. **c** Illumination spectra of 12-LED illuminator after bandpass filtering of each LED.

Manual imaging of multiplex IHC specimens was performed on an Olympus BX-51 microscope (Olympus, Waltham, MA) fitted with a CoolSNAP ES2 CCD camera with 1392 × 1040 pixels and 12-bit resolution (Teledyne Photometrics, Tucson, AZ) and Olympus UPlanSApo 20× (NA 0.75) and 10× (NA 0.40) air objectives. Illumination was provided by either optical filtering of a continuous light source or a 12-LED illuminator. For the former, a Sutter Lambda 10–3 10-position filter wheel (Sutter Instruments, Novato, CA) was used with an Olympus 100 W tungsten halogen lamp to define up to nine wavelength channels. Spectral characteristics (center transmission wavelength and FWHM) of six filters mounted in the filter wheel are listed in Table 1. The relative power of the tungsten lamp and each filtered light channel are plotted as a function of wavelength in Fig. 3a. The LED illumination comprised two Lumencor Spectra X light engines (Lumencor Inc, Beaverton, OR), each containing six custom-selected LEDs, the outputs of which were combined with dichroic beam splitters and focused onto a 3 mm diameter liquid light guide. The 3 mm guides from each light engine were combined into a single 3 mm diameter liquid light guide using a Lumencor combiner, and the light guide connected to the illumination port of the microscope through a Lumencor collimator/microscope adapter. To reduce further the illumination bandwidth, each LED was filtered with a single bandpass optical filter, the spectral characteristics of which are listed in Table 2. The relative output power of each LED versus wavelength is plotted in Fig. 3b, c for unfiltered and filtered LED outputs, respectively. Bandpass filters used with the LEDs and the filter wheel were obtained from Chroma Technology (Bellows Falls, VT) and IDEX Health and Science, LLC (Rochester, NY). Micromanager software was used to control acquisition of the CCD images of individual microscope fields [10].

**Table 1:** Normalized extinction coefficients for various chromogens determined for each light channel plotted in Fig. 3a, using filtered tungsten lamp illumination. Coefficients are scaled to 1.000 at the maximum absorbing light channel for each chromogen. Each light channel is designated by the center wavelength of the single bandpass filter and the filter FWHM.

Filter center λ (FWHM), nm	dabsyl	Rhod110	TAMRA	SRhod101	HTX	Cy5
438 (29.5)	1.000	0.220	0.078	0.177	0.374	0.050
510 (15)	0.606	1.000	0.553	0.373	0.483	0.089
549 (17.6)	0.203	0.494	1.000	0.688	0.663	0.274
580 (21.2)	0.114	0.210	0.487	1.000	0.849	0.523
620 (19.3)	0.077	0.129	0.057	0.170	1.000	0.878
676 (39.9)	0.063	0.102	0.034	0.061	0.471	1.000

**Table 2:** Normalized extinction coefficients for various chromogens determined for each light channel plotted in Fig. 3c, using filtered LED illumination. Coefficients are scaled to 1.000 at the maximum absorbing light channel for each chromogen. Each light channel is designated by the center wavelength of the single bandpass filter and the filter FWHM.

Filter center λ (FWHM), nm	DABSYL	RH110	TAMRA	SRH101	HTX	Cy5
390 (22.4)	0.547	0.062	0.085	0.116	0.123	0.027
415 (15.3)	0.816	0.053	0.098	0.101	0.211	0.013
438 (29.5)	0.952	0.078	0.042	0.098	0.266	0.013
475 (33.7)	1.000	0.411	0.093	0.076	0.268	0.019
504 (17)	0.580	0.958	0.370	0.150	0.450	0.017
513 (22.4)	0.444	1.000	0.481	0.213	0.439	0.024
549 (20.8)	0.080	0.156	1.000	0.565	0.749	0.060
580 (21.2)	−0.018	0.042	0.506	0.985	0.804	0.174
605 (22)	0.002	0.025	0.029	1.000	1.000	0.373
620 (19.3)	0.017	0.046	0.018	0.167	0.918	0.420
660 (20.2)	0.005	0.024	0.005	0.006	0.520	1.000
689 (29.5)	0.000	0.029	0.007	0.001	0.136	0.132

The automated pulsed illumination microscope system was constructed by retrofitting a Zeiss AXIO microscope (White Plains, NY) with the custom 12-LED illumination source described above and a Falcon2 8 MPx CMOS monochrome camera operating with 8 bits of resolution and 3328 by 2502 pixels (Teledyne DALSA, Waterloo, ON, Canada). The microscope utilized Zeiss Plan-APOCHROMAT 20× (NA 0.8) and 10× (NA 0.45) air objectives. The complete system was controlled using custom-developed hardware to enable rapid and repeatable image capture. The electronics to control the illumination pulses consisted of an Arduino Mega 2560, which connected to a PC that ran the user interface and camera image acquisition on one side, and discrete TTL logic that provided input/output line buffering for protection of the Arduino circuitry. The PC communicated the exposure times and wavelength sequence to the Arduino via USB communication and the Arduino then controlled the critical timing sequence between the Lumencor illuminators and the monochrome CMOS detector autonomously while the PC processed the images received through the framegrabber card (Dalsa Xcelera-CL PX4 Dual) in parallel. The automated system was capable of acquiring images of a microscope field illuminated sequentially with all 12 LEDs and transferred to the computer in ~700 ms.

### Characterization of automated imaging system

**Fig. 4:**
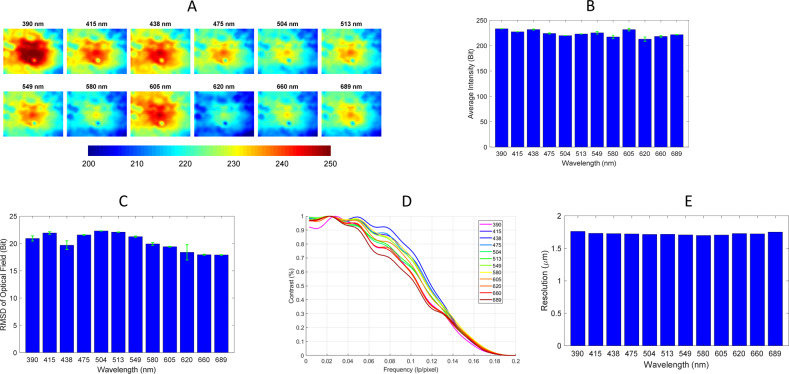
Characterization of automated rapid brightfield multispectral imaging system. **a** Illumination pattern on detector for all 12 wavelengths. **b** Average intensity of a blank image with automatically determined exposure times. **c** Root-mean-square-deviation (RMSD) of illumination field. **d** Modulation transfer function (MTF) of all wavelengths. **e** Spectral dependence of resolution as determined from the 3% cutoff frequency of the system’s MTF. Error bars represent standard deviation of 100 image acquisitions.

Prior to applying the automated system to multiplexed IHC of tumor specimens, the optical performance was characterized because each LED output is uniquely transmitted through the optical train. Initially, the unobstructed optical transmission profile was measured for all 12-LED wavelengths by imaging a blank location of the slide and acquiring 100 images at each wavelength. The average spatial profile of the illumination pattern was calculated and is displayed in Fig. 4a. It was also important to have near equivalent light across all wavelengths. An algorithm was written to iteratively increase each LED’s exposure time until the intensity was near the maximum of the detector to fully utilize the dynamic range of the system, but also with very few saturated pixels. As can been seen, the average intensity of the LEDs varies only between 213 bits at 620 nm and 233 bits at 390 nm after the algorithm adjusted each exposure time for consistent illumination intensities. Exposure times varied significantly from 3 ms at 549 nm to 166 ms at 689 nm, primarily because each LED had significantly different output power. It can be seen in Fig. 4b that shot-to-shot intensity of the LED illumination is very low with an average variation across all wavelengths of only 1.5 bits of intensity. From a signal-to-noise and repeatability standpoint, it is desirable to have as flat a field as possible, although nonflat fields are largely compensated for by dividing the tissue images with a blank image. Nonetheless, the flatness of each field was assessed by quantifying the root-mean-square deviation of each flat field as a surrogate of the overall profile of the light intensity; results are depicted in Fig. 4c. The complete optical system results in illumination patterns with roughly 15–20 bits of variation. Most importantly, the illumination profile was very consistent across many different LED pulses. A knife-edge test was performed to determine the modulation transfer function (MTF) of the system. An image was recorded of a sharp edge at each wavelength to calculate the line spread function (LSF). The LSF was then differentiated to calculate the point spread function (PSF) of the system. The spectral MTF was calculated by taking the Fourier Transform of each wavelength’s PSF. Finally, the resolution of the system was characterized at 20× by calculating when the MTF function drops to 3% contrast. Differences in the MTF can been seen in Fig. 4d. In particular, the far-red channels have slightly reduced contrast. However, this did not negatively impact the resolution, which was determined to be highly consistent at ~1.7 μm (i.e., 6 pixels) across all wavelengths, as seen in Fig. 4e.

### Imaging and image processing

When manually imaging a histological sample, the sample was sequentially illuminated with up to 12 different light channels (filter wheel plus tungsten lamp or LEDs) and images of the transmitted light acquired for each light channel. For automated imaging, a multispectral image cube was rapidly acquired by sequentially illuminating the tissue sample at each of the 12-LED wavelengths and recording an image for each. For manual and automated imaging, a blank image was also acquired at a clear location (no tissue) on the glass slide, representing 100% light transmission at each pixel for each light channel. Each tissue image was then divided by its corresponding blank image to calculate images of the fraction of light transmitted (transmission), which also served to compensate for the varying illumination pattern at the image plane both spectrally and spatially. Transmission images were log transformed to absorbance (A), used here as equivalent to optical density (OD), and spectrally unmixed using non-negative least squares to calculate the relative amount of each targeted protein [11, 12]. Absolute protein quantification is difficult in IHC without a well-characterized system with internal controls. Therefore, mappings of the relative amount of each protein were normalized for subsequent downstream image processing, such as measurements of selected areas and average and median pixel intensities.

Spectral unmixing requires *a priori* knowledge of each chromogen’s relative absorbance spectrum. Since chromogen spectra are affected somewhat by deposition, the on-slide absorbance of each chromogen at each illumination channel were determined. To accomplish this, IHC staining was performed separately for each chromogen on sections of tonsil tissue targeting Ki-67 and images recorded for each light channel. Median absorbance values were measured for the targeted regions using a mask generated on the image for which the particular chromogen absorbs maximally. Masks defined pixels with intensities above a threshold value that delineates the stained regions. Median absorbance values for different light channels were normalized to the median absorbance of the most strongly absorbing channel for each chromogen. The resulting normalized extinction coefficients recorded using the tungsten lamp and the LEDs are listed in Tables 1 and 2, respectively. These coefficients are similar to the values plotted in Fig. 2 using a spectrometer but account for the dye absorbance and illuminator wavelength dependence within the width of each light channel. For a particular multiplex IHC, the coefficients for the chromogens and light channels used in that multiplex form a matrix of coefficients, the inverse of which provides the correction coefficients for unmixing the observed absorbance images and converting to the relative biomarker abundance mappings. The images of relative biomarker abundance (relative concentration, proportional to OD) were then used to generate pseudo-color renditions of the assay by assigning each analyte a unique color in the RGB color space. When rendering pseudo-color images, each analyte concentration was normalized to a maximum of one to achieve color balancing. Reverse log transformation of rendered composite image planes provided brightfield-like representation. Images were also typically gamma corrected to accurately display linear concentration values. Image processing and color renditions were performed in MATLAB (Mathworks, Natick, MA, USA) and ImageJ [13]. Spectral unmixing using non-negative least squares was implemented in MATLAB.

## Results

### Chromogens and matching illumination channels

Five CDCs with relatively narrow absorbance bands spanning the wavelengths between 400 and 700 nm were selected for study in multiplex IHC. Absorbance spectra plotted in Fig. 2 show absorbance FWHM ranging from 139 nm for dabsyl to between 65 and 80 nm for the other four chromogens. Also plotted is the absorbance spectrum of the common nuclear stain, hematoxylin, which displays a broad FWHM of 192 nm, typical of conventional histology stains and chromogens. As one source of multispectral illumination, single bandpass interference filters were selected that aligned with the chromogen and hematoxylin absorbance bands, and used with the common tungsten halogen microscope lamp (Fig. 3a). Additional filters were selected for the purpose of oversampling the spectral information in a multiplex IHC specimen, and for accommodating future chromogens. With these filters at wavelengths above 400 nm, the manual CCD camera exposure times were typically 2 ms for the various tungsten lamp light channels, using neutral density filters between OD = 0.85 to 0.25 to maintain exposure times above a millisecond. LEDs, individually filtered with single bandpass filters to limit the breadth of each channel’s illumination, were evaluated as a second source of light channels (Fig. 3c). The 12-LED light channels are plotted over the chromogen spectra in Fig. 2, depicted as rectangular regions with widths equal to each LED filter’s FWHM. CCD camera exposure times were maintained at several milliseconds per light channel, using neutral density filters and LED input power.

Selecting the three broadly spaced chromogens plotted in Fig. 2, dabsyl, TAMRA, and Cy5, provides peak absorbance spacings of 100 nm. The spectral overlaps at these spacings, measured as crosstalk coefficients (see “Materials and Methods” section and Tables 1 and 2), range between 0.03 and 0.27, for neighboring chromogens, with an average coefficient of 0.13. Increasing the number of chromogens in a multiplex IHC to five, by adding Rhod110 and SRhod101, leads to chromogen absorbance peak separations between 35 and 65 nm, with an average spacing of 50 nm. At this closer spacing, overlap of the absorbance spectra is more significant, ranging between 0.03 and 0.69, for neighboring chromogens, with an average coefficient of 0.39. Addition of the hematoxylin nuclear stain further reduces the separation, and hematoxylin staining was decreased to minimize interference with neighboring chromogens.

### Multiplexed IHC with multispectral imaging—Manual control

**Fig. 5:**
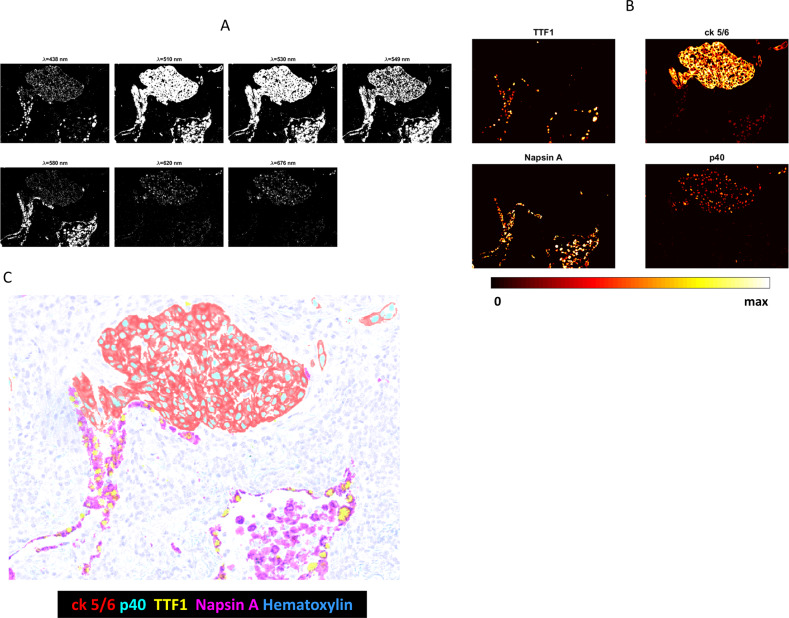
NSCLC 4-plex (plus hematoxylin nuclear stain) designed to differentiate squamous cell carcinoma (CK5/6+ and p40+) from adenocarcinoma (TTF1+ and Napsin A+). **a** Images of light absorbed (absorbance) using filtered tungsten lamp for illumination. **b** Unmixed images of each analyte with intensities mapped to colors according to the scale below the images. **c** Pseudo-colored brightfield image rendered from the unmixed images.

Multiplex IHC was performed on FFPE NSCLC tumor sections. The multiplex was designed to distinguish SCC from Ad Ca [9]. Antibodies to p40 and CK5/6, stained with the Cy5 and Rhod110 CDCs, respectively, were used to identify SCC, and antibodies to TTF-1 and Napsin-A, stained with dabsyl and TAMRA CDCs, respectively, were used to identify Ad Ca. Figure 5a shows seven absorbance images of light acquired by illumination with the five filters centered at 438, 510, 549, 620, and 676 nm, which provide the closest spectral alignment with the absorbance bands of dabsyl, R110, TAMRA, hematoxylin, and Cy5, respectively, plus filters centered at 530 and 580 nm for oversampling. Figure 5b shows the four spectrally unmixed images corresponding to each biomarker’s relative abundance, in which values are mapped to colors according to the scale displayed below the images. The color composite image in Fig. 5c was formed by pseudo-coloring the unmixed images of each biomarker and hematoxylin to re-create a brightfield visual representation. Pseudo-coloring to mimic visual observation was employed showing the purple TAMRA and yellow dabsyl staining of the biomarkers indicative of Ad Ca, and the red-orange Rhod110 and blue-green Cy5 staining of the biomarkers indicative of SCC. For exemplary purposes, the specimen selected for Fig. 5 is a mixed SCC and Ad Ca (adenosquamous) tumor.

**Fig. 6:**
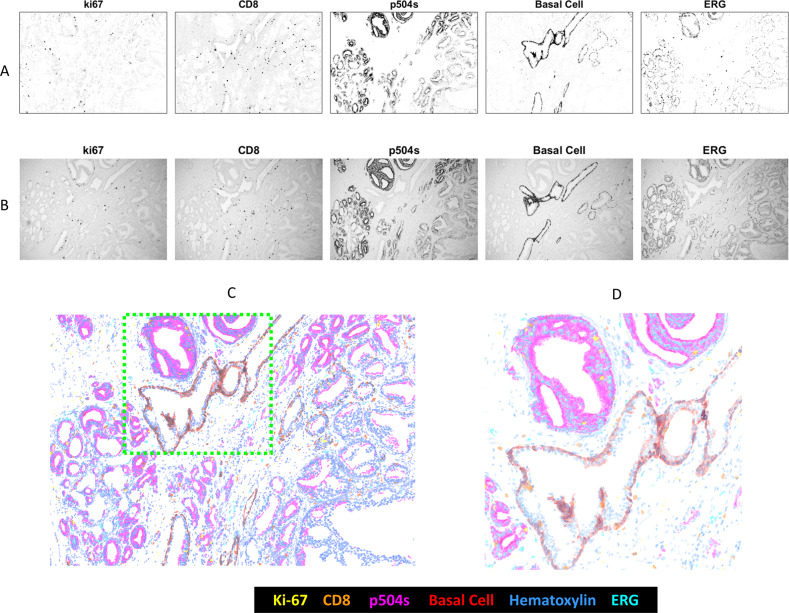
Prostate 5-plex (plus hematoxylin nuclear stain). **a** Unmixed images of each analyte’s relative abundance, converted to brightfield representations for comparison to DAB-stained serial sections. **b** Images of DAB stain for each individual protein targeted in the multiplex assay on separate serial sections. **c** Pseudo-colored brightfield image rendered using unmixed analyte abundances. **d** Zoomed view of portion of (**c**) indicated with dashed box.

Multiplexing was increased to five CDCs, with the addition of the SRhod101 CDC, and applied to FFPE prostate tumor sections. Transmitted light images were recorded using the five filters centered at 438, 510, 549, 580, and 676 nm aligning with the dabsyl, Rhod110, TAMRA, SRhod101, and Cy5 CDCs directed to regions expressing Ki-67, CD8, P504S (AMACR), basal cell marker (cocktail of antibodies to p63 and keratin), and ERG, respectively. An image using the filter centered at 620 nm was recorded for the hematoxylin counterstain. Figure 6a displays the five CDC images after unmixing and conversion to brightfield representations. DAB chromogenic staining (transmitted light) for the same five expressed markers on sequential FFPE sections are presented in Fig. 6b for comparison of staining patterns. The color composite image, also in a brightfield representation, is shown in Fig. 6c, with an expanded region shown in Fig. 6d. Pseudo-coloring intentionally differs from the coloring observed under the microscope in order to improve visualization.

The multi-spectral imaging system was able to resolve unmixed analyte concentrations with good accuracy. For example, the average absolute error between the experimentally obtained images and spectrally unmixed images presented in Fig. 5 for the NSCLC specimen was only 0.06 absorbance units, which is an error of 1.9% relative to the assay’s maximum absorbance. As expected, the majority of that error was in the center wavelengths (*λ* = 510–580 nm) where chromogen spectral cross-talk was significantly higher than in the blue and red parts of the spectrum where dabsyl and Cy5, respectively, can be imaged with minimal spectral overlap. Measured error was even less in the prostate 5-CDC multiplex, at about half that of the NSCLC multiplex.

### Multiplexed IHC with rapid brightfield multispectral imaging

**Fig. 7:**
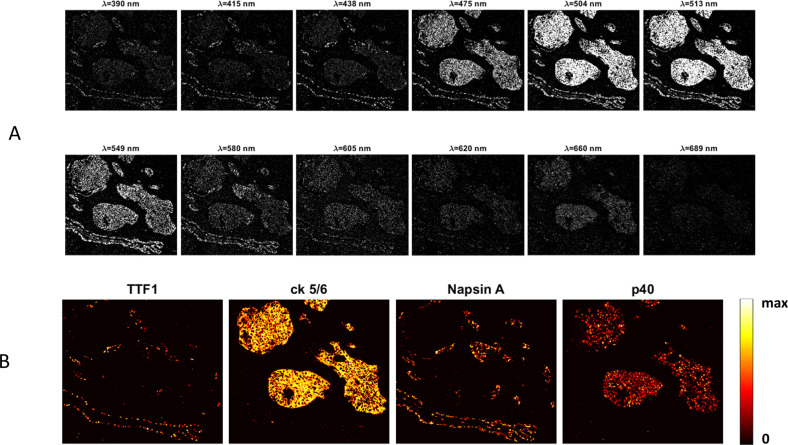
Rapid brightfield multispectral imaging of NSCLC 4-plex (plus hematoxylin nuclear stain) with 12-LED illuminator. **a** Multispectral images of light absorption (optical density). **b** Unmixed images of each biomarker with intensities mapped to colors according to the colorbar.

**Fig. 8:**
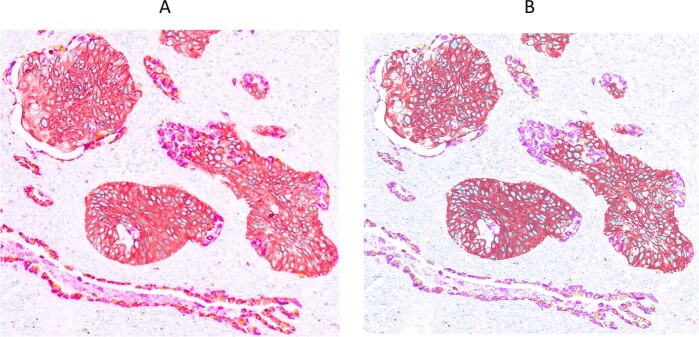
NSCLC multiplex assay recorded with color camera compared with multispectral composite image. Comparison of color images of NSCLC specimen as (**a**) recorded on a standard RGB camera and (**b**) a pseudo-colored visualization formed from the unmixed multiplex images with colors chosen to match the assay as viewed through the microscope ocular.

The automated multispectral imaging system was tested on the same stained adenosquamous NSCLC tumor specimen analyzed previously (Fig. 5). Transmitted images were recorded in rapid succession for each of the 12-LED channels as shown in Fig. 7a, converted to OD. Spectral unmixing produced biomarker abundance images, displayed in Fig. 7b. Figure 8 presents a direct comparison of the NSCLC assay as viewed through an ocular (i.e., as recorded on a standard RGB color camera), shown in Part A, versus a composite color image constructed from spectrally unmixed chromogen images recorded with the pulsed LED system and pseudo-colored to resemble the ocular view, showing near equivalence.

### Processing of multispectral composite images for improved interpretation

**Fig. 9:**
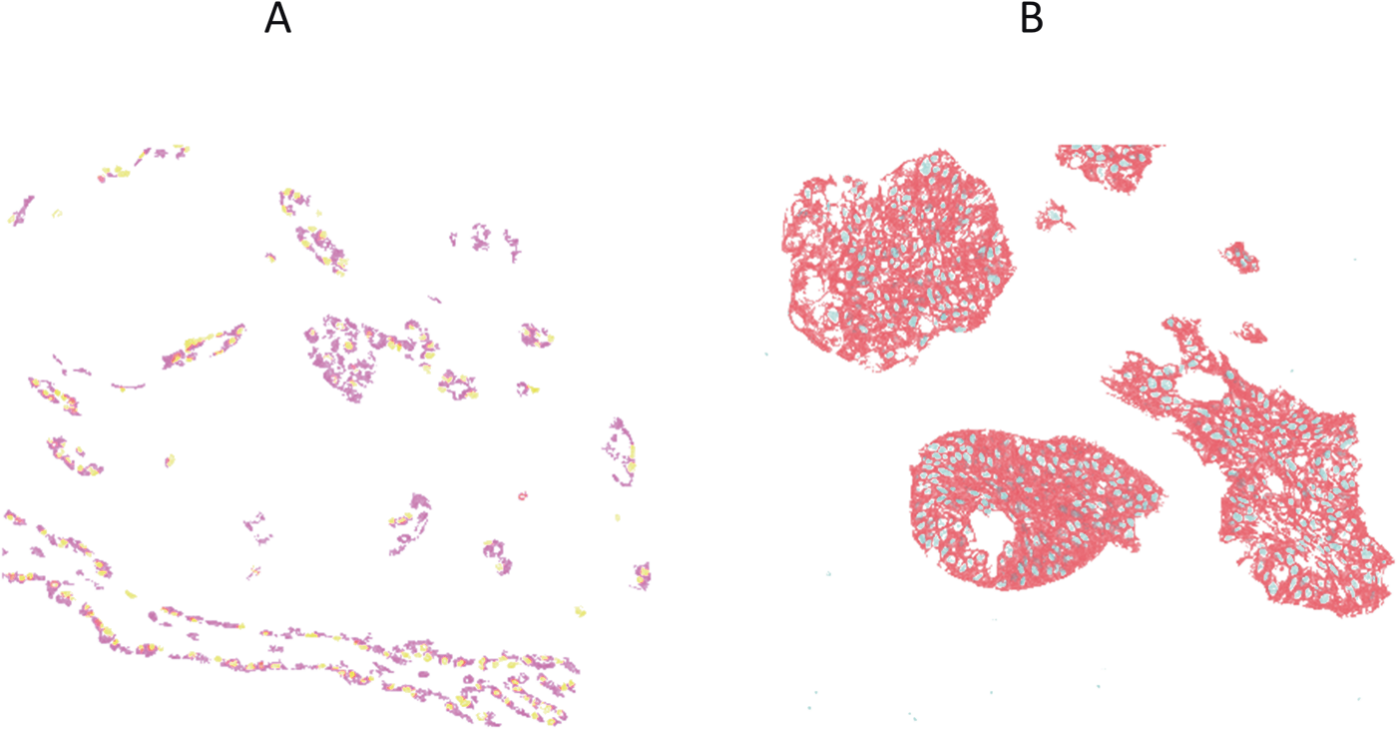
Color composite images showing only two biomarkers each, to reduce complexity and improve visual interpretation. **a** Visualization of NSCLC assay shown in Fig. 8b with only Ad Ca markers present (Napsin A and TTF1). **b** Visualization of NSCLC assay shown in Fig. 8b with only SCC markers displayed (CK5/6 and p40).

**Fig. 10:**
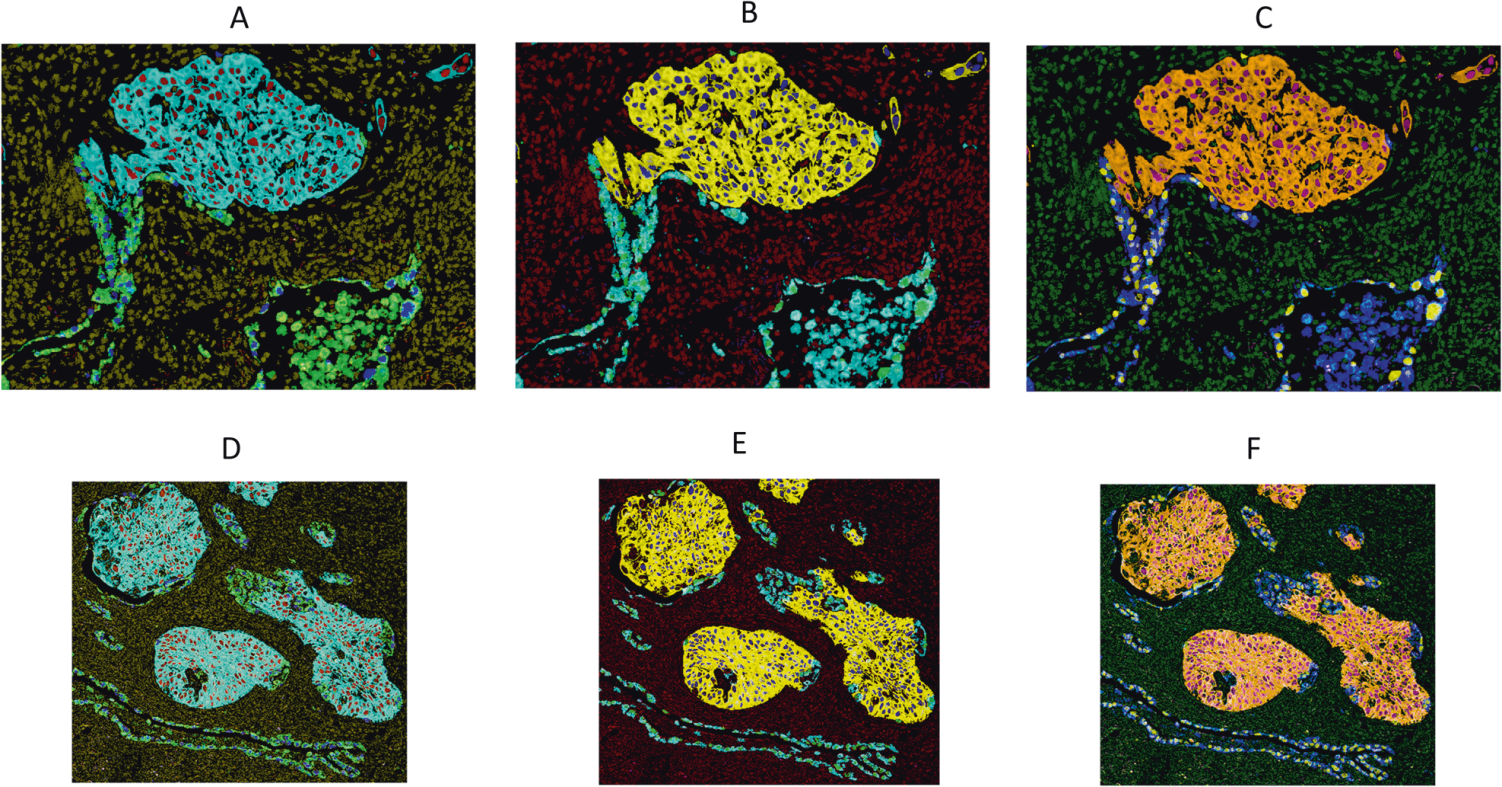
Color composite images depicting flourescent-like visualizations of NSCLC multiplex assays. Examples of different pseudo-color renderings of fluorescence-like representations of NSCLC assays displayed in Figs. 5c and 8b.

The unmixed relative biomarker abundance images for a multiplex IHC may be used directly for quantitative analysis, since the pixel values are proportional to the analyte concentration, assuming a constant amplification factor for chromogen deposition, the same as for fluorescence images from IF. Public access software is available for quantitative processing (e.g., ImageJ) as well as commercial software (e.g., Matlab), and a number of high-level software packages are specifically aimed at analyzing biological and clinical specimens (e.g., Indica Labs, Albuquerque, NM; Visiopharm, Westminster, CO; Definiens, Carlsbad, CA). The unmixed relative abundance images are used also to build color composite images for visual interpretation and may be rendered in many ways. These include controlling the number of expressed proteins that are visible in any one composite, as shown in Fig. 9. The image in part A only displays the 2 protein markers associated with Ad Ca and the right image only displays the SCC markers. Figure 10 parts A–C and parts D–F shows how the brightfield color composite representations presented in Fig. 5 and Fig. 8b, respectively, can be presented in fluorescence-like representations and how pseudo-coloring can be used to tailor images to personal preference.

## Discussion

Brightfield microscopy of multiplex IHC, as implemented here, matches chromogens possessing narrow absorbance bands with spectrally aligned illumination channels and monochrome detection. This combination provides a path to higher level multiplexing than previously permitted in brightfield multiplex IHC. CDCs afforded a number of narrow absorbance band chromogens from which to choose and light channels were created either by a filter wheel combined with a continuous emission microscope lamp or by a collection of LEDs. Both types of light channels provided monochrome images that could be adequately unmixed and combined into color composite images in 5-plex (four chromogens plus hematoxylin nuclear stain; Figs. 5, 7, and 8), and 6-plex (five chromogens plus hematoxylin nuclear stain; Fig. 6) IHC.

When designing multiplex assays, proper selection of chromogens and illumination channels is important to limit spectral crosstalk to levels that unmixing algorithms can faithfully correct. The spectral crosstalk of neighboring dyes is evident in the absorbance images before any crosstalk correction in Fig. 5a. Crosstalk is particularly noticeable for the 510 nm, 530 nm, and 549 nm light channels where absorbance of both the Rhod110 and TAMRA chromogens is evident in all three images. The corresponding unmixed images in Fig. 5b clearly show the effective removal of crosstalk, revealing the separation of all four chromogens, with an unmixing error of about 2%. This indicates that all biomarkers in the assay are well distinguished from one another. Furthermore, this approach is scalable to higher order multiplexing with narrow absorbance band chromogens and optimal chromogen spacing. Currently we have prepared and characterized 15 CDCs with different absorbance maxima and peak widths from which to select for assembling multiplex assays. The development of new chromogen is an ongoing effort to enlarge the pool of candidate chromogens for increased multiplexing capacity. Minimizing crosstalk and unmixing errors is critical to decreasing the detection limits in multiplex IHC, and is an important consideration in continuing work. Future work is needed to characterize the effect of deposition density on reference spectra shape as well as the long-term stability of chromogen absorption profiles. A thorough evaluation of optimal unmixing algorithms and techniques is also needed to mature the technology. Despite these potential improvements, in our lab we have found chromogen spectra to be stable for multiple months. We recalibrate our reference spectra only when necessitated by a chemistry or hardware change to the imaging platform.

An important question to answer in multiplexed assays is whether the simultaneous staining of multiple biomarkers affects the observed expression patterns of the biomarkers. This question was addressed in the prostate 6-plex assay by performing “gold standard” DAB IHC for each of the five biomarkers individually on prostate sections cut serially to the multiplex section. Comparisons of each unmixed biomarker image from the multiplex IHC (Fig. 6a) to the corresponding DAB images (Fig. 6b) show equivalent staining patterns signifying that the multiplex IHC faithfully reproduces biomarker expression. It is worth noting that the multiplex assays presented here do not include examples of co-expressed proteins. Chromogens deposited at the same location due to protein coexpression can have an effect on one another, with deposition of subsequent chromogens reduced by prior chromogen deposition. In addition, because this technology is based on absorption, multiple colocalized chromogens could be difficult to resolve if there is limited transmission at a particular wavelength. To fully characterize the technology, future work will include assessing the optical detection limits of the approach and quantifying the number of colocalized chromogens that can be resolved.

An important advantage of brightfield multiplexing is the speed of image acquisition. The 12-LED illumination source coupled with a conventional brightfield microscope and monochrome CMOS camera, as described here, was capable of high-speed image acquisition, with most individual LED illumination times of several milliseconds or less and 8 Mpixel frame transfer times of 11 ms. Image acquisition and storage could be achieved in 700 ms. This time includes considerable oversampling for the assay shown here and reduction of oversampling will proportionately provide further time reduction. For 20× magnification, whole-slide scanning (1.5 cm × 1.5 cm) with rapid 12-LED sequential illumination is expected to be several times longer than a conventional brightfield scanner. Image processing to unmix chromogens and recombine to form color composite images extends these times, and minimal processing time is being addressed in future work. This approach may enable whole slide scanning with scan times considerably shorter than current fluorescence scanners.

Characterization of the optical system for rapid acquisition showed good and consistent performance. The average spatial profile of the illumination pattern was similar for all 12 LEDs and pulse-to-pulse intensity variation for each LED was small. An algorithm written to control LED exposure time ensured each channel fully utilized the system’s dynamic range while minimizing saturated pixels. While calculation of the transmission image itself serves to flatten each field, assessment of flatness by root-mean-square-deviation of each original field showed only small variation in light intensity, and resolution was highly consistent across all wavelengths.

The unmixed images resulting from a chromogenic multiplex IHC are suitable for quantitative analysis using algorithms similar to those applied to multiplex IF. As shown in Figs. 9 and 10 they can also be combined in different ways to produce color composite images that enhance visual interpretation. Simultaneous viewing of multiple colored stains in a composite can be confusing, particularly as multiplexing increases beyond three analytes. An effective approach to simplification is to allow the user to select the number and identity of the biomarkers seen at one time, as shown in Fig. 9 in which only the two Ad Ca (left) or two SCC (right) markers in the adenosquamous NSCLC specimen are visible at the same time. Pseudo-coloring as shown in Figs. 5c, 6c, 8b, and 10 can effectively differentiate multiple markers, and color selection can help compensate for differing color acuity and preferences between users. Finally, conversion from brightfield representation to fluorescence-like representation, as shown in Fig. 10, can provide further visual detail and accommodate different user preferences.

While the work here primarily establishes a multiplexing technology, this work also demonstrates clinical value. The multiplex IHC of four chromogens plus hematoxylin nuclear stain in Fig. 5 clearly shows that both Ad Ca and SCC are present in this tumor. IHC is not always needed to identify Ad Ca and SSC, with standard H&E staining often being adequate, but a significant number of cases do benefit from the additional IHC definition, and knowing the tumor type is important prognostic and treatment-related information [9, 14]. Adenosquamous tumors can be particularly difficult to discern since they are less common and one tumor type may dominate. The presence of both tumor types is immediately obvious when all four markers are used simultaneously [9]. The multiplex IHC of Fig. 6 provides important information relative to prostate cancer diagnosis and prognosis. The basal cell marker is important in histologically suspicions lesions since prostate carcinoma lacks basal cells [15]. Similarly, the presence of P504S can be important in establishing malignancy [16]. Ki-67, an indicator of growth, CD8, identifying activated T-cells, and EGR, associated with tumor aggressiveness [17], can provide important ancillary and prognostic information.

A concern of brightfield IHC relative to IF is the perceived higher sensitivity and dynamic range of IF. While it is true that fluorescence in pristine solutions is orders of magnitude more sensitive than absorbance, clinical sensitivity is determined on biological specimens of complex composition. The inherent greater dynamic range of fluorescence is reduced by unavoidable fluorescence background. Intra-tissue and tissue-to-tissue stain variability, as particularly affected by pre-analytics that affects both IF and brightfield measurements, further erodes the signal-to-noise advantages of IF microscopy. In the future, multiplex IHC and IF on clinical cohorts with known patient outcomes will be necessary to compare meaningfully the clinical sensitivity and specificity between the two techniques.

In summary, this work establishes feasibility of higher-order brightfield multiplexing using multispectral imaging. This work overcomes the major limitations of brightfield multiplexing by utilizing narrowband chromogens and matching illumination channels with monochrome optical detection. This novel approach is simultaneously capable of generating high-quality images compatible with decision support algorithms as well as brightfield visualizations of the assay that a pathologist would be capable of using for diagnosis or quality control. This feature will help make pathologists more comfortable with a digital solution and promote widespread clinical adoption. In addition, this technology benefits from simplified optics and significantly faster scan times than an IF-based scanner, which will facilitate improved clinical workflow and encourage uptake of a digital pathology system. This technology addresses many of the barriers that have prevented clinical adoption of IHC multiplexing, and with further refinement may expedite clinical adoption by combining the rich medical value of multiplex diagnostic assays with the speed, pathologist familiarly, and broadly established clinical utility of brightfield microscopy.
